# Application of synthesized copper nanoparticles using aqueous extract ofZiziphus mauritiana L. leaves as a colorimetric sensor for the detection of Ag ^+^

**DOI:** 10.3906/kim-2001-51

**Published:** 2020-10-26

**Authors:** Roomia MEMON, Ayaz Ali MEMON, Syed Tufail Hussain SHERAZI, Sirajuddin Sirajuddin, Aamna BALOUCH, Muhammad Raza SHAH, Sarfaraz Ahmed MAHESAR, Kausar RAJAR, Muhammad Hassan AGHEEM

**Affiliations:** 1 National Centre of Excellence in Analytical Chemistry, University of Sindh, Jamshoro Pakistan; 2 International Center for Chemical and Biological Sciences, HEJ Research Institute of Chemistry, University of Karachi, Sindh Pakistan; 3 Center for Pure and Applied Geology, University of Sindh, Jamshoro Pakistan

**Keywords:** Copper nanoparticles, *Ziziphus mauritiana* L., plant leaves extract, sensor, silver ion

## Abstract

The presented work demonstrates the preparation of copper nanoparticles (CuNPs) via aqueous leaves extract of *Ziziphus mauritiana* L. ( *Zm* ) using hydrazine as a reducing agent. Various parameters such as volume of extract, concentration of hydrazine hydrate, concentration of copper chloride, and pH of the solution were optimized to obtain *Ziziphus mauritiana* L. leaves extract derived copper nanoparticles ( *Zm* -CuNPs). Brownish red color was initial indication of the formation of *Zm* -CuNPs while it was confirmed by surface plasmon resonance (SPR) band at wavelength of 584 nm using ultraviolet-visible (UV-vis) spectroscopy. Synthesized *Zm* -CuNPs were characterized by Fourier transform infrared spectroscopy (FT-IR), scanning electron microscopy (SEM), atomic force microscopy (AFM), and X-ray diffractometry (XRD). AFM images showed that the particle size of *Zm* -CuNPs was from 7 to 17 nm with an average size of 11.3 nm. Fabricated sensor ( *Zm* -CuNPs) were used as a colorimetric sensor for the detection of Ag ^+^ at a linear range between 0.67 × 10 ^-6^ – 9.3 × 10 ^-6^ with R ^2^ value of 0.992. For real water samples, limit of quantification (LOQ) and limit of detection (LOD) for Ag ^+^ was found to be 330 × 10 ^-9^ and 100 × 10 ^-9^ , respectively.

## 1. Introduction

Silver (Ag) is a rare, naturally occurring element in the earth. It is considered as one of the more important metals after gold, for the preparation of ornaments. It is widely used in a variety of objects, especially in jewelry and tableware. It has versatile nature and exhibits many industrial and medicinal properties, such as, being a catalyst, used in photography, brazing alloys, electrical conductors, batteries, imaging, silver solder, biomedical, dental alloys, pharmaceutical, and antibacterial activities [1–4]. However, some adverse effects have been reported on the human body due to the use of silver in the dental amalgam, catheters, accidental wounds, and needles [5]. Additionally, high intake of silver may lead to blood pressure, stomach irritation, decreased respiration, and increase in diarrhea [6], whereas, prolonged ingestion of low doses of silver may produce fatty liver and kidney disease [7]. Similarly, argyrosis disease is also caused by long term ingestion of soluble silver compounds, as their small amount accumulates in the brain and muscles. Decrease in mitochondrial functions, organ failure and cytotoxicity may also be due to the chronic exposure of silver ions (Ag ^+^ ) [4]. Therefore, the maximum permitted level of silver in drinking water by Environmental Protection Agency of US is 0.9 µM [5]. Assessment of silver is very important in marine ecosystems for environmental monitoring and public health.[6]. Negative impacts of silver on human health are still controversial and are not very well established [7]. However, it is well known fact that high amounts of silver have adverse effects on aquatic life and are considered as one of the main environmental pollutants [8]. Consequently, determination of Ag ^+^ at trace level in water has remained a very important task for many researchers for health and economical reasons. Already, various techniques such as inductive couple plasma mass spectrometric methods (ICP-MS) [9], stripping voltammetry [10], atomic emission and electrochemical methods [11], and stripping and Kelvin force probe microscopic methods [12] have been reportedly used for the detection of silver ion at trace level. But these methods require lengthy sample preparation, hazardous chemicals, sophisticated instruments, and need trained operators. To overcome these issues, optical sensors based on metal nanoclusters for determination of silver ion have been developed due to cost-effectiveness, simple, and quick observation [13]. Colorimetric method for the detection of Ag ^+^ at trace level has also been reported by many researchers [1, 14, 15]. Research is based on continuous improvements. From an economic and an environmental point of view, a favorable method for the synthesis of nanoparticles may include working at room temperature, at neutral pH, and green reducing and capping materials. Generally, plants are considered as natural “chemical factories”. It has been confirmed through various studies that the reduction of metals into their respective metals nanoparticles has been carried out through plant extracts containing polyphenols, terpenoids, alkaloids, sugars, proteins, and phenolic acids. A variety of plant extracts have been used for green synthesis of different metal nanoparticles such as cobalt [16], Ag [17], Au [18], Pd [19], ZnO [20], magnetites (Fe and Ni) [21–22], and Cu NPs [23–26]. *Ziziphus mauritiana* L. ( *Zm* ) is a fruit tree and well known for its medicinal as well as nutritional benefits [27,28]. It is commonly known as Jujube and locally known as ‘Ber’. It is a tropical fruit found in many parts of the world including Pakistan, Africa and India. It belongs to the family *Rhamnaceae* [29]. It has multiple medical advantages like antihyperglycemic, antiinflammatory, antiplasmodial, and antimicrobial activities, as well as hemolytic anemia, sedative (tranquilliser), anxiolytic, diuretic, analgesic (pain reliever), and antioxidant properties. The leaves of *Zm* are are also very beneficial to human health and are eaten with catechu as an astringent. They are considered as diaphoretic and especially preferred for typhoid in children [10].

In the current study, the synthesis of CuNPs involves aqueous leaves extract of *Zm* and hydrazine hydrate as a reducing, as well as, an oxygen removing agent. Fabricated copper nanoparticles (CuNPs) were used as a colorimetric sensor for the detection of silver (Ag ^+^ ) at trace level in real water samples. As we know, there are the two novel aspects of the current work; it is the first time that *Zm* plant extract has been used for the synthesis of copper nanoparticles, and there are currently no other studies on copper nanoparticles for colorimetric sensing of Ag ^+^ .

## 2. Experimental

### 2.1. Chemicals and reagents

In the current study, all chemicals and reagents were of analytical grade and used without any further treatment. Copper chloride (CuCl _2_ .2H _2_ O), hydrazine monohydrate (N _2_ H _4_ .H _2_ O 99.9%), silver nitrate (AgNO _3_ 99.9%), potassium nitrate (KNO _3_ 99.9%), zinc chloride (ZnCl _2_ 97%), calcium chloride (CaCl _2_ 97%), cadmium chloride (CdCl _2_ 98 %), lead chloride (PbNO _3_ 99.5%), sodium nitrate (NaNO _3_ 99%), magnesium chloride (MgCl _2_ 99.9%), hydrochloric acid (HCl 37%), nitric acid (HNO _3_ 98%), sodium hydroxide pellets (NaOH 99%), and ethanol (C _2_ H _5_ OH 97%) were obtained from Sigma-Aldrich Corp. (St. Louis, MO, USA). The preparation of solution was carried out by dissolving a specific amount of each chemical in Milli-Q water (EMD Millipore Corp., Billerica, MA, USA).

### 2.2. Instrumentation

Ultraviolet-visible (UV-vis) absorption spectra of synthesized *Zm* -CuNPs were recorded on Lambda 356 spectrophotometer (PerkinElmer Inc., Waltham, MA, USA) between 200–800 nm. Interaction between CuNPs and phytochemicals of plant extract was confirmed by FT-IR spectrophotometer (Nicolet 5700 of Thermo Madison, Thermo Electron Scientific Instruments Corp., Madison, WI, USA). Scanning electron microscopy (SEM JSM-6380 LV, JEOL Ltd., Tokyo, Japan) was used to analyze the structural characterization and morphology of prepared *Zm* -CuNPs. To confirm the size and shape of NPs, atomic force microscope (Agilent 5500, Agilent Technologies, Inc. Santa Clara, CA, USA) was used. Crystalline properties of fabricated *Zm* -CuNPs were confirmed by XRD (D-8, Bruker AXS GmbH, Karlsruhe, Germany). Digital camera was used to record visual colorimetric detection of Ag ^+^ by copper nanoparticles.

### 2.3. Preparation of plant leaves extract

Fresh leaves (25 g) of *Ziziphus mauritiana* were weighted and added into 100 mL volumetric flask. The solution was boiled at 100 °C for 15 min then cooled at room temperature. Whatman filter paper (No.1) was used for filtration of the extract to get a clear solution. The filtrate was stored at 4 °C for further synthesis of nanoparticles.

### 2.4. Synthesis protocol of copper nanoparticles

Various parameters, such as, volume of aqueous extract of Zm leaves, volume of reducing agent (1 M hydrazine solution), volume of precursor salt (0.01 M CuCl _2_ .2H _2_ O), and pH were optimized by UV-vis spectrometer and the data represented in the supplementary file as Figures S1–S4, respectively.

**Figure 1:**
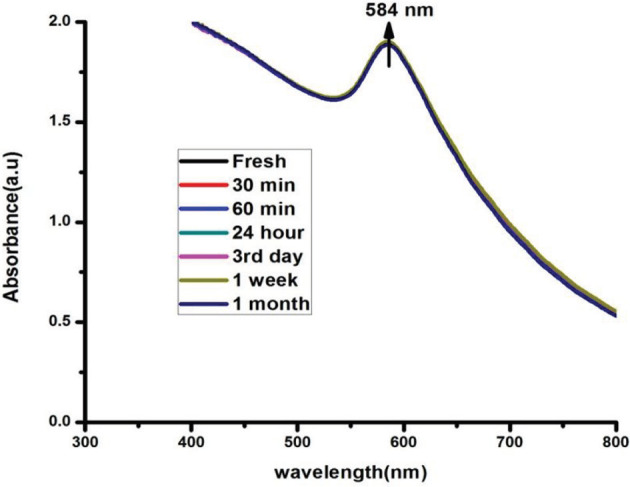
Time based stability of synthesized Zm-CuNPs.

As per optimization study, 1 mL of 0.01 M CuCl _2_ .2H _2_ O, 0.5 mL of plant extract, and 2 mL of 1 M hydrazine hydrate were added into a 10 mL test tube and filled with Mili-Q water up to the mark at neutral pH. The solution mixture was left at room temperature until it appeared brown in color, which indicated the successful formation of *Zm* -CuNPs. No stirring or heating was involved in the process. UV-vis spectroscopy was used for initial confirmation of the formation and stability of the *Zm* -CuNPs.

### 2.5. Procedure for colorimetric sensing of silver ion

The colorimetric detection of silver ion was carried out at room temperature. For the development of calibration, known concentration of Ag ^+^ ranging from 0.67 to 9.3 µM prepared from silver nitrate stock solution (0.1 μM) mixed with 3 mL of biosynthesized copper nanoparticles solution. After a few minutes the solution was transferred into a 1 cm quartz cell to check colorimetric response. Spectroscopic study was conducted at spectral range from 200 to 800 nm by using Milli-Q water as reference reagent. Change in color from brown to blackish color was considered as a visual check and change in absorbance (delta absorbance) during LSPR study and was used as a quantitative response for the determination of silver in colorimetric system. Color change was observed from brown to blackish with change in absorbance from lower to higher against a blank solution for calibration. The analogous color alterations were also achieved with digital camera after reaction time and it was compared with previously reported detection of silver by other nanoparticles.

### 2.6. Preparations of real water samples

Different types of water samples such as tape water, surface water, and other sources from different localities were collected, filtered, and diluted to the desired volume. Various concentrations of analyte (Ag ^+^ ) were spiked from stock solution (0.1 μM) into 3 mL of *Zm* -CuNPs. The solutions were kept for 3–4 min at room temperature and then data recorded for each sample in triplicate with the help of UV-vis spectrophotometer.

## 3. Results and discussion

### 3.1. UV-visible spectroscopy

In the last few decades, several studies reported that the optical response of metal nanoparticles (NPs) can be adjusted to control size and shape of synthesized nanoparticles [23]. Localized surface plasmon resonance (LSPR) modes of metallic nanoparticles (such as copper, silver, and gold) exist in the visible region of the electromagnetic spectrum. UV/Vis spectroscopy was used for the optimization of different parameters such as precursor salt (CuCl _2_ ), reducing agent (hydrazine hydrate), capping agent (leaves extract), and pH for the synthesis of small size CuNPs.

Effect of volume of aqueous extract of *Ziziphus mauritiana* leaves on λ _max_ of CuNPs is shown in Figure S1.

Different volumes ranging from 0.5 to 3 mL of *Ziziphus mauritiana* ( *Zm* ) leaves extract were used to obtain the optimum volume on the basis of blue shift which indicated smaller size of copper nanoparticles. The best result was achieved by using 0.5 mL of plant extract ( *Zm* ) at a wavelength of 590 nm by keeping the amount of precursor salt and reducing agent constant. CuNPs have an affinity to oxidize immediately in aqueous medium which is a major negative aspect for using these particles as colorimetric sensors. Hence, to provide an inert environment and to stabilize CuNPs, hydrazine hydrate was used as a reducing agent, which resists the oxidation of CuNPs by evolving nitrogen. Results show the change in LSPR band from 590 nm to a hypsochromic shift of 587 nm by using 1 mL of hydrazine hydrate 0.5–3 mL keeping the constant volume of *Zm* plant extract. The effect of the volume of 1 M hydrazine solution on λ _max_ shift of CuNPs is shown in Figure S2.

Effect of volume of precursor salt (0.01 M CuCl _2_ ) ranging from 0.5 to 3 mL solutions is shown in Figure S3. The bathochromic shift and precipitation occurred with bigger particle size using an increased quantity of precursor salt (3 mL). This change in LSPR band may be result of an increased rate of nucleation with greater quantity of copper II ions present in solution. However, 1 mL of precursor salt was selected for further studies.

pH is an important factor for the stability of nanoparticles. Figure S4 shows pH effect on the blue shift, λ _max_ , and shape of the peak, which is related to the size of copper nanoparticles in the pH range between 4 and 10.

Furthermore, various factors such as the size of nanoparticles, agglomeration, and nature of capping agents also play important roles in the position, shape, and size of LSPR band. Conversely, protonation/deprotonation of acidic group present in *Zm* -CuNPs might be followed due to change in pH of solution. Substantial changes occurr in shape and width of LSPR band as pH increases from 4 to 10 and pH 7 was selected as optimum pH for *Zm* -CuNPs on the basis of blue shift and shape of SPR band from broad to narrow.

Figure 1 shows UV-visible spectra of synthesized CuNPs with respect to the stability. No significant change was observed in either the color, or in the wavelength of colloidal solution with passage of time. The results show that synthesized *Zm* -CuNPs under optimized parameters were found to be stable for upto 1 month. Therefore, fabricated *Zm* -CuNPs could be used as sensing probe during a wider period and could be stored at room temperature without using special storage conditions.

### 3.2. Fourier transform infrared spectroscopy

**Figure 2:**
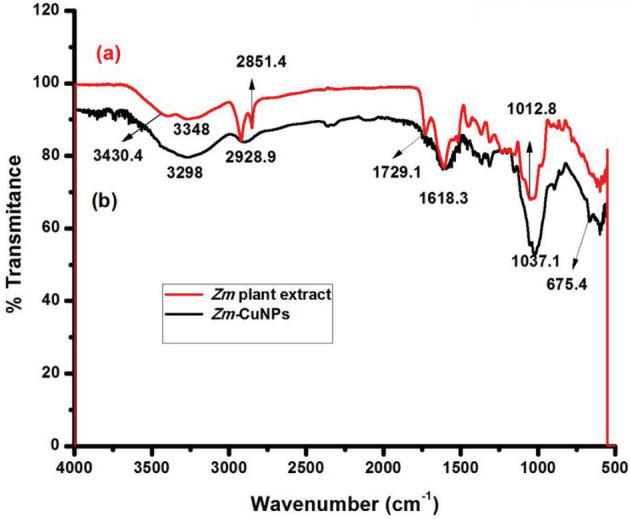
FTIR Spectrum of (a) Zm leaves extract (b) Zm capped CuNPs.

FTIR technique was used to observe interaction between CuNPs and biomolecules of plant extract. Figure 2a shows the FTIR spectrum of plant material and Figure 2b shows the spectrum of *Ziziphus mauritiana* extract capped CuNPs. Bands at 3430.4 cm ^-1^ and 3348 cm ^-1^ in the FTIR spectrum of the leaves extract are shifted to 3298 cm ^-1^ in the FTIR spectrum of *Zm* -CuNPs. Moreover, band at 1729.1 cm ^-1^ is due to carbonyl group present in the plant extract (Figure 2a) which has disappeared in FTIR spectrum of *Zm* -CuNPs (Figure 2b). There is also a band at 1618.3 cm ^-1^ due to NH bending of amide group in both spectra. Also, by comparing the fingerprint region, a new signal at 674.5 cm ^-1^ was observed in FTIR spectrum of *Zm* -CuNPs due to the presence of CuNPs, as it is not present in FTIR spectrum of leaves extract. Therefore, absence of carbonyl band of leave extract and appearance of new peak at 675.5 cm ^-1^ in FTIR spectrum of *Zm* -CuNPs indicated that interaction of biomolecules of leaves extract occurred through carbonyl band with CuNPs.

### 3.3. Scanning electron microscopy (SEM)

**Figure 3:**
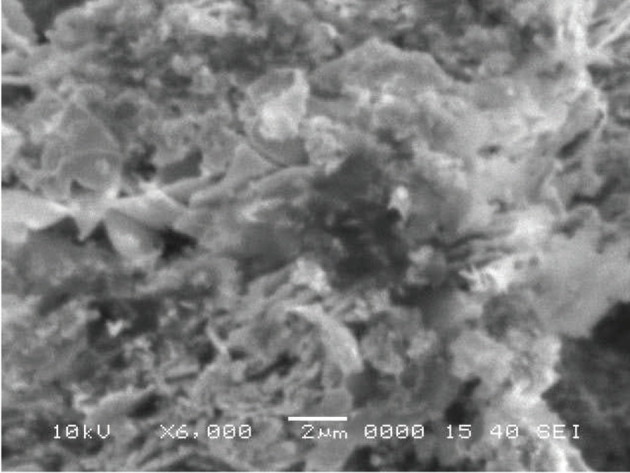
SEM image of Zm-CuNPs.

Surface morphology of Zm-CuNPs was studied by SEM image of *Zm-* CuNPs (Figure 3). It was observed that NPs have a rough surface with a spongy, flower like shape. Greater catalytic activity may be due to roughness of surface of *Zm* -CuNPs with larger surface areas [30].

### 3.4. Atomic force microscopy (AFM)

**Figure 4:**
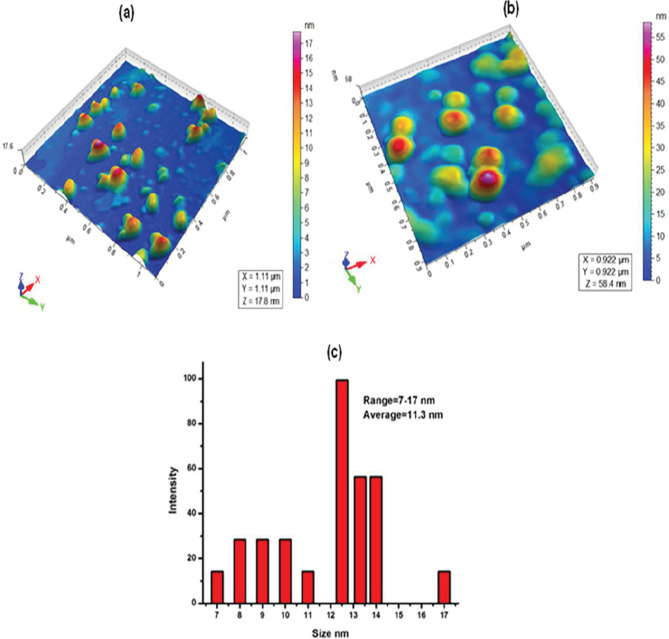
AFM images of Zm-CuNPs (4a) before sensing of Ag ^+^ (4b) after addition of Ag ^+^ (4c) size distribution histogram for Zm-CuNPs.

AFM technique offers visualization and analysis of nanomaterial in three dimensions. As per AFM images (Figure 4a), *Zm* -CuNPs ranged between 7 and 17 nm with an average size of 11.3 nm which was calculated by ImageJ software. Figure 4b shows that size was increased after addition of silver into synthesized *Zm* -CuNPs up to 55 nm. Before sensing *Zm* -CuNPs were monodispersed and spherical in shape as shown in Figure 4a but after the addition of Ag ^+^ , the morphology and size of *Zm* -CuNPs were totally altered as shown in Figure 4b, may be due to the formation of alloy of Cu and Ag [31]. Figure 4c shows size distribution histogram for *Zm* -CuNPs on the basis of data achieved from AFM.

### 3.5. X-ray diffractometry (XRD)

**Figure 5:**
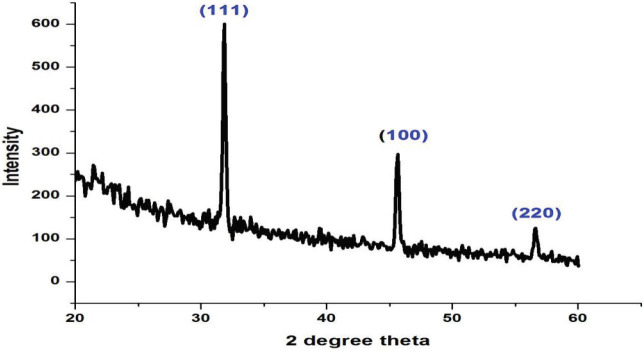
Diffraction patterns of Zm-CuNPs with face center cubic planes (FCC) at (111), (100), and (220).

X-ray powder diffraction (XRD) is a rapid analytical technique basically used for phase recognition of a crystalline material and can provide information on unit cell dimensions. Figure 5 indicates that the diffraction pattern of *Zm* -CuNPs was distinctive face center cubic (FCC) planes of CuNPs at (111),(100), and (220) with high crystalline level at 2θ angles of 31.7°, 45.3°, and 56.4° respectively. These standard planes at particular angles prove that *Zm* -CuNPs are crystalline in nature, verified with the JCPDS data (card no. 89‑5899). XRD pattern is comparable with the already reported study [32].

### 3.6. Colorimetric sensing of Ag ^+^

**Figure 6:**
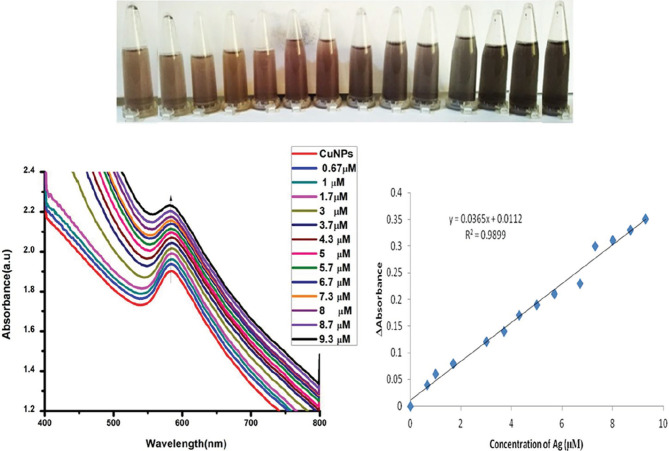
(a) UV-visible spectra of Zm-CuNPs with different concentration of Ag ^+^ (0.67–9.3 × 10 ^–6^ M) while inset shows color change with respective addition of Ag ^+^ (b) Linear regression plot of added concentration of Ag ^+^ (μM) to Zm-CuNPs versus change in absorbance (ΔA).

Figure 6a illustrates colorimetric performance of Zm-CuNPs after the addition of different concentrations of Ag ^+^ in the range between 0.6 × 10 ^-6^ – 9.3 × 10 ^-6^ M using UV-vis spectroscopy. Calibration curve showing increase in absorbance with increasing concentration of Ag ^+^ from 0.67–9.3 × 10 ^-6^ M, inset shows color change with respective addition of Ag ^+^ . The color change was observed gradually from brown to blackish after each addition of Ag ^+^ . Figure 6b shows the linear plot of added Ag ^+^ concentration in µM versus ∆ absorbance. The LOD and LOQ values for Ag ^+^ were found to be 100 × 10 ^-9^ ,and 330 × 10 ^-9^ M, respectively. LOD was determined as (3*σ)/ slope of linear plot while LOQ was determined as (10*σ)/ slope of linear plot; σ is denoting the standard deviation of at least 3 blank runs measured in ∆ absorbance value.

### 3.7. Selectivity of sensor

**Figure 7:**
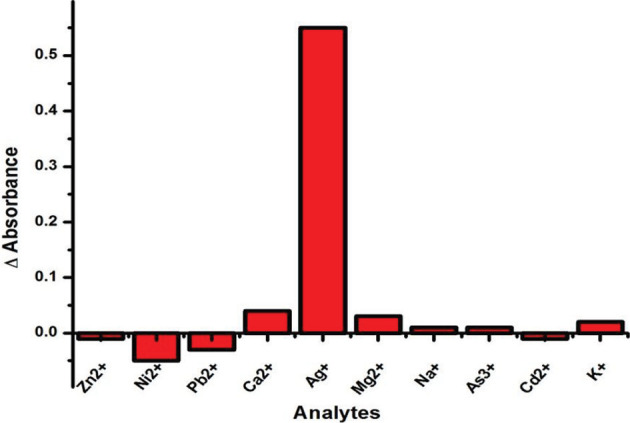
Selectivity of the proposed sensor for Ag ^+^ detection in the presence of possible interfering ions.

Selectivity of colorimetric sensor for Ag ^+^ in the presence of Zn ^2+^ , Ni ^2+^ , Pb ^2+^ , Ca ^2+^ , Mg ^2+^ , Na ^+^ , Cd ^2+^ , As ^3+^ , and K ^+^ at the concentration of 10 µM was evaluated. The absorption intensity was examined under the same experimental conditions for other metal ions. From Figure 7, it is very clear that no substantial decrease of the absorption signal was observed in the presence of tested interfering ions. The results clearly indicate that there is no significant effect on absorbance and color change of *Zm* -CuNPs solution upon the addition of other tested metal ions, except silver ion which showed distinctive color change with the change in absorbance of surface plasmonic resonance (SPR) band.

### 3.8. Figures of merit

Comparative results of the currently developed colorimetric sensor with already reported studies [12,14,33–36] are illustrated in Table 1. Although, AuNPs were successfully applied as sensors for the detection of Ag ^+^ , the good ranges but the use of Au salts makes these sensors highly expensive. Therefore, developed *Zm* -CuNPs based Ag ^+^ sensor is highly sensitive as LOD is much lower than most of the reported sensors. All the reported colorimetric sensors for the detection of Ag ^+^ are based on AuNPs. According to our best knowledge, our work is the first report of using CuNPs for Ag ^+^ detection. Moreover, *Ziziphus mauritiana* plant extract was used as capping agent which is easily available, cheap, and many active bio-molecules have been already reported in *Ziziphus mauritiana* plant extract [29].

**Table 1:** Figures of merit of reported and present studies using various metal nanoparticles for detection of Ag ^+^ in water.

Method	Probe	Linear range (M)	LOD (M)	Reference
Colorimetric	BSA@AuNCs	0.5–1 × 10 ^–6^	0.204 × 10 ^–6^	[14]
Colorimetric	AuNPs	1–9 × 10 ^–6^	0.41 × 10 ^–6^	[33]
Colorimetric	AuNPs	0.01–1 × 10 ^–6^	7.4 × 10 ^–6^	[34]
Colorimetric	AuNPs	5–40 × 10 ^–6^	1 × 10 ^–6^	[35]
Colorimetric	AuNPs	0.1–4 × 10 ^–6^	0.05 × 10 ^–6^	[12]
Colorimetric	AuNPs	2–28 × 10 ^–6^	0.85 × 10 ^–6^	[36]
Colorimetric	CuNPs	0.6–9.3 × 10 ^–6^	0.1 × 10 ^–6^	Current work

### 3.9. Comparative studies

Table 2 shows that aqueous extract of different plants has been reported for preparation of various nanoparticles [22,37–44]. In the present study, aqueous leaves extract of *Zm* plant was used as a green material for the synthesis of CuNPs. A small size of Zm-CuNPs ranging from 7 to 17 nm was achieved, compared to reported studies.

**Table 2:** Different plants used for synthesis of copper nanoparticles by different researchers.

Plant	Precursor salt	Particle size (nm)	Reference
Ocimum sanctum	CuSO _4_	8–140	[22]
Nerium oleander	CuSO _4_	40–100	[37]
Punica granatum	CuCl _2_	40–80	[38]
Eclipta prostrate	Cu(CH _2_ COO) _2_	28–45	[39]
Punica tenuiflorum	CuSO _4_	56–59	[40]
Asparagus adscendens	CuSO _4_	50–65	[41]
Aloe vera	CuSO _4_	15–30	[42]
Hemidesmus indicus	CuSO _4_	26–30	[43]
Allium sativum	CuSO _4_	83–130	[44]
Ziziphus mauritiana	CuCl _2_	7–17	Current work

### 3.10. Detection of Ag ^+^ from different real water samples

For practical application of synthesized plant extract based CuNPs sensor, a river water sample was collected from river Indus, Pakistan along with some tape water samples. Ag ^+^ was determined by standards addition method. Six known concentrations of Ag ^+^ (3, 4, and 5 µM in river water and 2, 7, and 9 μM in tape water) were prepared using spiking protocol. The desired volume of each standard was mixed with *Zm* -CuNPs solution and 3 replicate runs were recorded for each analysis. Detection of spiked Ag ^+^ in real water samples with percentage recovery is demonstrated in Table 3. The result revealed that Ag ^+^ in real water sample was successfully detected by *Zm* -CuNPs as a colorimetric sensor with recovery between 96.2 and 102.5%.

**Table 3:** Detection of spiked Ag ^+^ in real water samples with percentage recovery.

Samples	Actual (µM)	Spiked (µM)	Found (µM)	SD (±)	% Recovery
S-1	0	3	2.96	0.02	98.6
S-2	0	4	4.10	0.01	102.5
S-3	0	5	4.81	0.01	96.2
Tape water					
S-4	0	2	1.98	0.03	99.0
S-5	0	7	6.90	0.50	98.5
S-6	0	9	8.89	0.01	98.7

## 4. Conclusion

We conclude that the present work focuses on a new strategy with a greener, cheaper, and facile way of producing highly stable *Zm* -CuNPs using a newer capping agent from *Ziziphus mauritiana* leaves extract. These synthesized nanoparticles are highly stable for up to one month at room temperature and neutral pH (7), without the need of any inert environment. This synthetic strategy is highly economical, more simple, efficient, and less time consuming. Moreover, these stable *Zm* -CuNPs were applied as a sensitive, selective, and economical colorimetric sensor for detection of silver ion at micro molar level concentration. The best merit of the study lies in the fact that highly stable CuNPs are due to strong capping potential of phytochemicals of plant extract with a small size (11.3 nm), compared to other reported studies.
